# NAD^+^ loss, a new player in AhR biology: prevention of thymus atrophy and hepatosteatosis by NAD^+^ repletion

**DOI:** 10.1038/s41598-017-02332-9

**Published:** 2017-05-23

**Authors:** Silvia Diani-Moore, Jenny Shoots, Rubi Singh, Joshua B. Zuk, Arleen B. Rifkind

**Affiliations:** Department of Pharmacology and Pharmacology PhD Program, Weill Cornell Medicine, 1300 York Avenue, NY, NY 10021 USA

## Abstract

Dioxin (2,3,7,8-tetrachlorodibenzo-*p-*dioxin, TCDD) is a carcinogenic and highly toxic industrial byproduct that persists in the environment and produces a pleiotropic toxicity syndrome across vertebrate species that includes wasting, hepatosteatosis, and thymus atrophy. Dioxin toxicities require binding and activation of the aryl hydrocarbon receptor (AhR), a ligand activated transcription factor. However, after nearly 50 years of study, it remains unknown how AhR activation by dioxin produces toxic effects. Here, using the chick embryo close to hatching, a well-accepted model for dioxin toxicity, we identify NAD^+^ loss through PARP activation as a novel unifying mechanism for diverse effects of dioxin *in vivo*. We show that NAD^+^ loss is attributable to increased PARP activity in thymus and liver, as cotreatment with dioxin and the PARP inhibitor PJ34 increased NAD^+^ levels and prevented both thymus atrophy and hepatosteatosis. Our findings additionally support a role for decreased NAD^+^ dependent Sirt6 activity in mediating dioxin toxicity following PARP activation. Strikingly, treatment *in vivo* with the NAD^+^ repleting agent nicotinamide, a form of vitamin B3, prevented thymus atrophy and hepatosteatosis by dioxin and increased sirtuin activity, providing a therapeutic approach for preventing dioxin toxicities *in vivo*.

## Introduction

Activation of the aryl hydrocarbon receptor (AhR), a ligand activated transcription factor, is required for production of toxic effects by a group of polyhalogenated hydrocarbons, a common class of environmental toxins of which dioxin (2,3,7,8-tetrachlorodibenzo-*p*-dioxin, TCDD) is the most notorious member^1, 2^. TCDD produces a toxicity syndrome in multiple species that includes wasting, thymus involution, hepatosteatosis, and dysregulated glucose metabolism^3–5^. Even after many years of study it remains unclear how TCDD produces its diverse spectrum of toxic effects. Activation of the AhR by TCDD increases transcription of a large number of genes^6^, none of which has yet been able to account for the production of toxic effects by TCDD in multiple organs *in vivo*.

Here we investigated whether NAD^+^ depletion has a role in TCDD toxicity *in vivo* based on our prior evidence from experiments conducted in cultured chick embryo hepatocytes (CEH) that TCDD decreased NAD^+^ levels while increasing expression of TiPARP (TCDD-inducible poly (ADP-ribose) polymerase, PARP7, ARTD14)^7^. TiPARP is one of 17 PARP enzymes that consume NAD^+^ while ADP-ribosylating proteins^8^ and an AhR target gene^7, 9–11^. We used thymus atrophy, an effect of TCDD in all vertebrate species studied^3, 12^, and hepatosteatosis, another common effect of TCDD^13–15^, as endpoints for TCDD toxicity. As thymus atrophy and hepatosteatosis are pathologically distinct we expected that identification of a mechanism that causes both thymus atrophy and hepatosteatosis would shed light on the basis for the diverse effects of TCDD. Using the chick embryo close to hatching, a well-accepted model for TCDD toxicity^3^, we identified NAD^+^ loss as a unifying mechanism for these TCDD toxicities. We show that TCDD *in vivo* decreases NAD^+^ levels in both thymus and liver and that it does so by increasing PARP activity. NAD^+^ loss in turn decreases Sirt6 activity in both organs, supporting a role for suppression of sirtuin activities in TCDD toxicities. NAD^+^ repletion with nicotinamide (NAM) or the PARP inhibitor PJ34 prevented both NAD^+^ depletion and the production of thymus atrophy and hepatosteatosis by TCDD, supporting a role for NAD^+^ loss in different TCDD toxicities *in vivo*.

## Results

### TCDD produces thymus atrophy and hepatosteatosis while decreasing NAD^+^ in thymus and liver; NAD^+^ repletion by nicotinamide prevents these effects

TCDD treatment activated the AhR in thymus as well as liver as evidenced by TCDD increasing mRNAs for AhR target genes *CYP1A4* and *TiPARP* in both organs (Supplementary Fig. S1). While *TiPARP* levels were higher at 3 hr than at 24 hr after TCDD treatment, *CYP1A4* induction continued to increase at 24 hr and remained sustained at 72 hr. Similar differences in the time courses of CYP1A and TiPARP induction in primary chick embryo hepatocytes and in T-47D cells were previously demonstrated^7, 16^.

**Figure 1:**
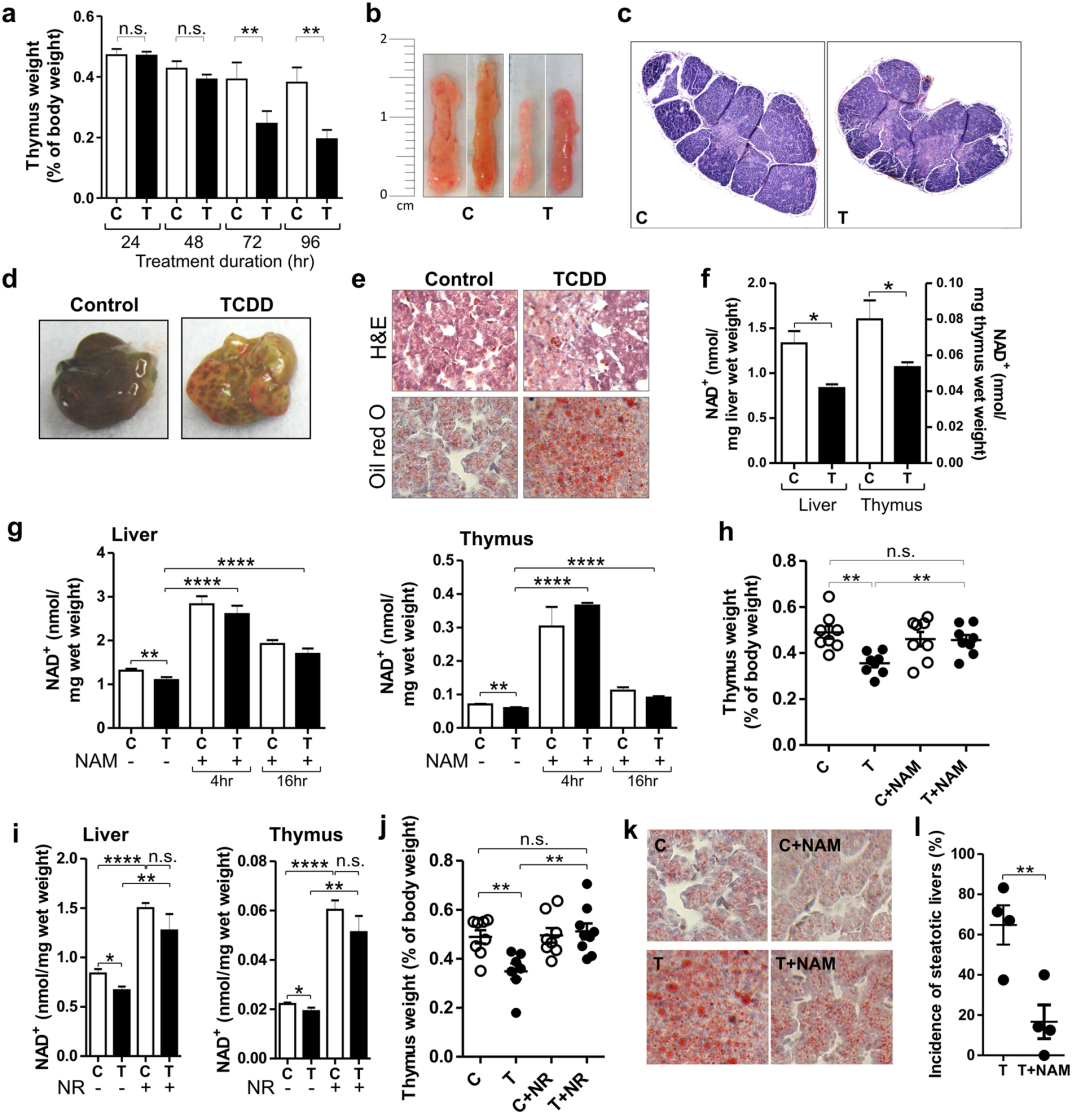
TCDD produces toxic effects while decreasing NAD^+^ levels. Prevention by NAD^+^ repletion. (**a**) Mean thymus weights as percentages of body weight ± SE for CE treated with TCDD (T) or dioxane (vehicle control, C). For 24 and 48 hr time points, n = 8 individual embryos. For the 72 and 96 hr time points, 4 independent experiments with n = 3–8 embryos per treatment group. (**b**) Photographs of representative CE thymus glands. (**c**) Representative CE thymus sections stained with H&E. (**d**) Representative livers from CE treated with TCDD or vehicle. (**e**) H&E and Oil Red O stained liver sections. (**f**) NAD^+^ levels in livers and thymus glands. (**g**) Mean NAD^+^ levels ± SE in livers and thymus glands of CE treated for 24 hr with TCDD with or without NAM during the last 4 or 16 hr of TCDD treatment (n = 3–8 CE per treatment group). (**h**) Scatter plot showing thymus weights as percentages of body weight for CE treated with TCDD or vehicle with and without NAM (n = 7–8 CE per treatment group) with means ± SE for each treatment group. (**i**) Mean NAD^+^ levels ± SE for livers and thymus glands from CE treated with TCDD (24 hr) with or without nicotinamide riboside (NR) for the last 4 hr. (**j**) Scatter plot showing means ± SE for thymus weight for CE treated with TCDD with or without nicotinamide riboside (NR) (n = 7–9 CE per treatment group). (**k**) Representative Oil red O stained sections of CE livers. (**l**) Scatter plot for 4 independent experiments, each with n = 7–10 CE per treatment group, showing the incidence of steatosis. Control livers lacked signs of steatosis. For all Figures: *p < 0.05; **p < 0.01; ***p < 0.001; ****p < 0.0001; n.s., not significant; TCDD treatment was for 72 hr unless indicated.

Treatment of chick embryos (CE) with TCDD decreased thymus weight significantly after 72 hr and even more after 96 hr (Fig. 1a,b and c) without affecting body weights over this time period. Occasional lymphocytolysis found in thymus cortex from TCDD treated CE supports a diagnosis of thymic atrophy rather than hypoplasia, consistent with TCDD effects in other species^12^.

TCDD produced steatotic changes in CE liver at the same dose and treatment duration that produced thymus atrophy (Fig. 1d). Necrotic (pale) and disorganized areas as shown by H&E staining and increased lipid droplets as evidenced by Oil red O staining were seen in liver sections from CE 72 hr after TCDD treatment (Fig. 1e). Supplementary Fig. S2 shows that the changes in Oil red O in Fig. 1 reflect changes in triglyceride levels. Hepatosteatosis and thymus atrophy by TCDD were accompanied by decreased NAD^+^ levels (TCDD decreased NAD^+^ by 38% (p = 0.02) in liver and by 33% (p = 0.03) in thymus (Fig. 1f)).

To explore a role for NAD^+^ depletion in the toxic effects of TCDD we treated CE with nicotinamide (NAM), the amide form of vitamin B3, which undergoes cellular metabolism to NAD^+^^17^, as a NAD^+^ repleting agent. A single dose of NAM increased NAD^+^ levels in thymus and liver for at least 16 hr (Fig. 1g). A second dose of NAM prolonged the elevation of NAD^+^ levels (Supplementary Fig. S3).

Along with preventing decreases in thymus NAD^+^ levels by TCDD, NAM treatment prevented thymus atrophy (Fig. 1h). Nicotinamide riboside (NR), a NAD^+^ repleting agent which is converted to NAD^+^ through a different pathway than NAM^18, 19^, also increased NAD^+^ levels in liver and thymus (Fig. 1i) and prevented thymus atrophy by TCDD (Fig. 1j). Neither TCDD nor NAD^+^ replenishing agents, NAM or NR, significantly increased *NAMPT* or *NMNAT1* mRNAs, suggesting that increased NAD^+^ levels by NAD^+^ replenishing agents were not dependent on changes in expression of NAD^+^ salvage pathway enzymes.

The ability of two different NAD^+^ repleting agents to prevent thymus atrophy strongly supports a role for NAD^+^ depletion in production of thymus atrophy by TCDD. NAM also diminished hepatic vacuolization and lipid accumulation by TCDD as shown by Oil Red O staining and triglyceride measurements (Fig. 1k and Supplementary Fig. S4) and the incidence of steatosis (Fig. 1l), supporting a role for NAD^+^ depletion in steatosis as well as thymus atrophy by TCDD.

We previously observed that NAM could inhibit AhR activation at high doses in cultured CEH^7^. Supplementary Fig. S5 shows that NAM did not inhibit CYP1A4 induction by the activated AhR *in vivo* at a dose that protected against thymic atrophy and hepatosteatosis, indicating that AhR activation by TCDD persisted in the presence of NAM *in vivo* under the conditions shown here.

### TCDD increases PARP activity in both thymus and liver

**Figure 2:**
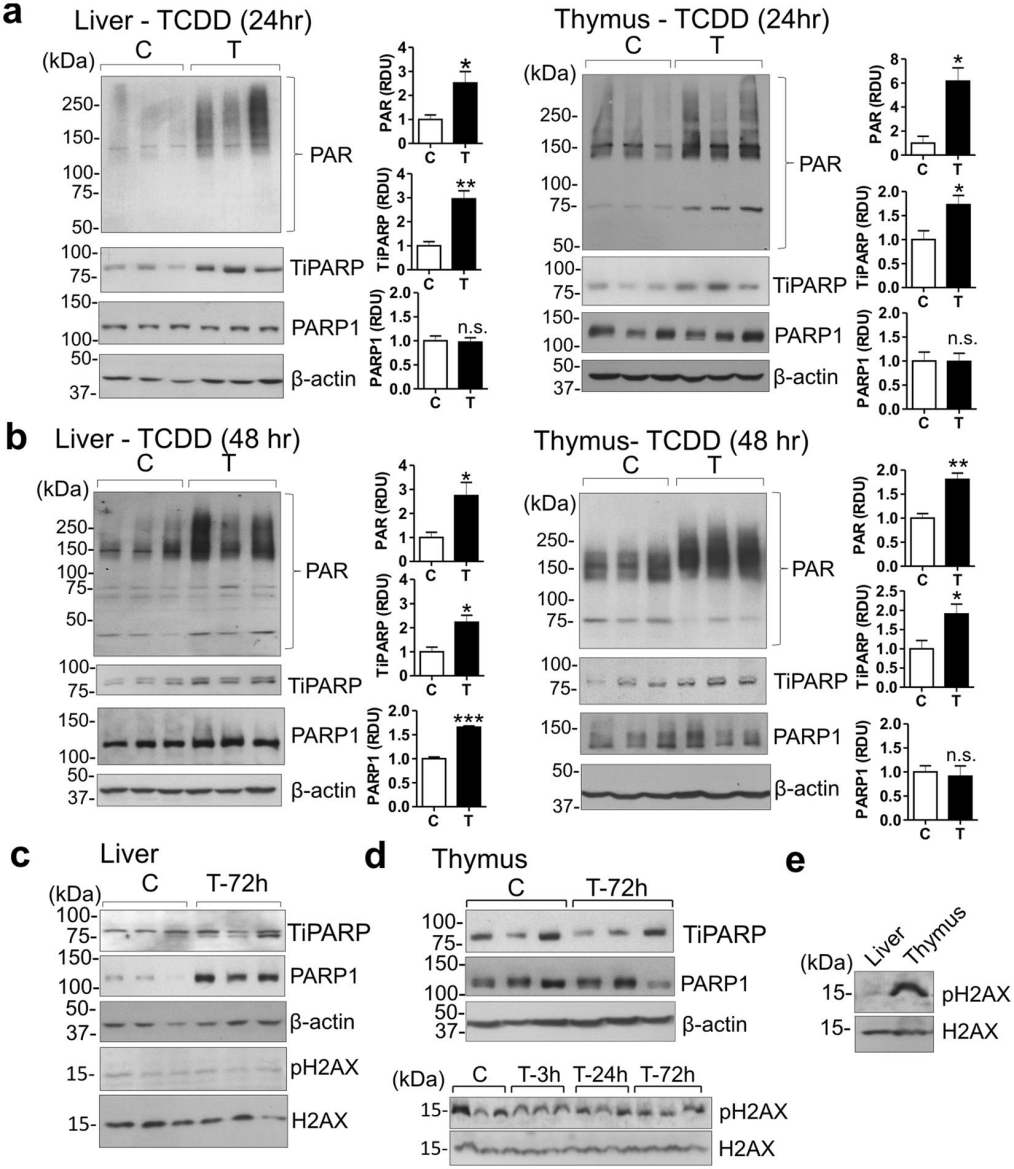
TCDD increases PARP activity *in vivo*. (**a–d**) Western blots on homogenates of liver and thymus from CE treated with TCDD (T) or vehicle (C). Bar graphs show means ± SE for relative densitometry units (RDU) for triplicate lanes. (**e**) pH2AX levels in liver and thymus from untreated CE. For western blots for CE homogenates in this and other Figures, each lane is from an individual CE.

As PARP enzymes consume NAD^+ ^^8, 20^ and TCDD increases PARP7 (TiPARP)^7, 9^, we asked whether suppression of NAD^+^ levels by TCDD *in vivo* might reflect increased PARP activity. Figures 2a and b show that TCDD increased PARP activity as indicated by western blotting with an anti-PAR (polyADP-ribose) antibody. PARP activity was evident in liver and thymus of the controls and along with TiPARP levels, was increased 24 and 48 hr after TCDD treatment in both organs. After 48 hr TCDD also increased levels of PARP1 protein (the major PARP enzyme responsible for more than 80% of PARP activity^8^) in liver but not in thymus (Fig. 2b). TiPARP induction was diminished in liver at 72 hr after TCDD treatment and was no longer elevated in the thymus glands at that time (Fig. 2c and d).

Hepatic PARP1 levels were further increased 72 hr after TCDD treatment as compared to PARP1 levels 24 hr after TCDD (Fig. 2c). TCDD increased PARP1 protein in liver without increasing *PARP1* mRNA (Supplementary Fig. S6), suggesting that TCDD may have increased PARP1 translation or stability rather than its transcription. While increased DNA damage is commonly associated with increased PARP1 activity, TCDD did not increase levels of pH2AX, an index of DNA damage^21^, in liver or thymus for up to 72 hr after treatment (Fig. 2c,d), excluding DNA damage as responsible for increased PARP1 in liver or thymus atrophy in TCDD treated CE. Fig. 2e shows that pH2AX levels in untreated CE were higher in thymus than liver.

Notably, PAR, TiPARP and PARP1 levels were higher in thymus than liver for both control and TCDD treated CE (Supplementary Fig. S7a). PAR levels were also higher in mouse thymus than liver (Supplementary Fig. S7b) indicating that the findings are applicable to a mammalian model. Supplementary Fig. S7b also shows also that PAR levels were higher in younger than in older mice.

### The PARP inhibitor PJ34 increases NAD^+^ levels and prevents toxic manifestations of AhR activation

**Figure 3:**
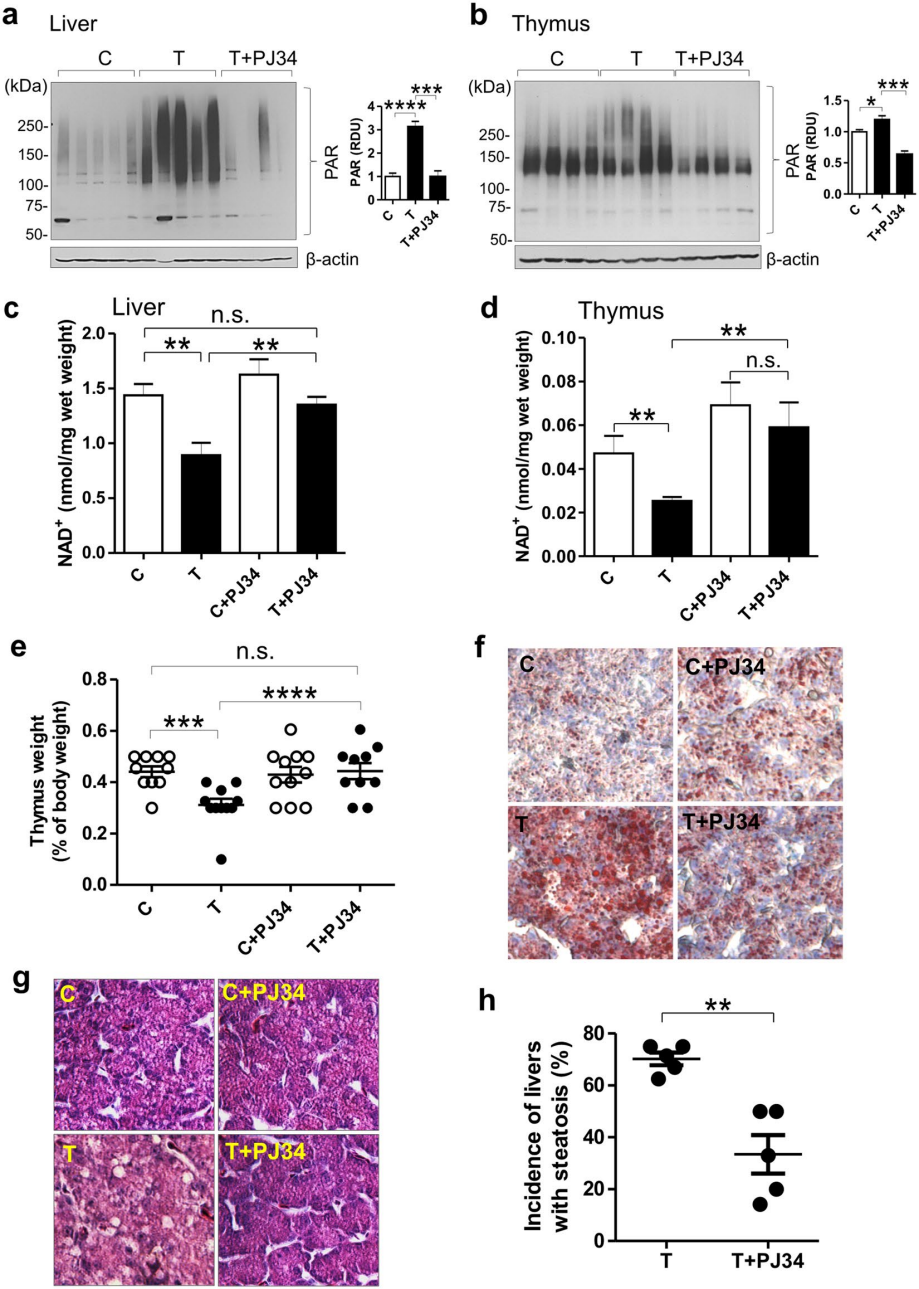
The PARP inhibitor PJ34 prevents TCDD toxicities while increasing NAD^+^ levels. **(a**,**b**) Western blots on homogenates of liver and thymus glands from CE treated with TCDD or vehicle with or without PJ34. Bar graphs show means ± SE for relative densitometry units (RDU) for the blots shown. (**c**,**d**) Mean NAD^+^ levels ± SE in CE homogenates of livers and thymus glands. (**e**) Scatter plot with means ± SE for thymus weights. (**f**) Representative Oil red O stained liver sections. (**g**) Representative H&E stained liver sections. (**h**) Scatter plot for 5 independent experiments showing the percentage of livers with steatosis (n = 10–12 CE per treatment group in each experiment). Control livers did not have any signs of steatosis.

Treatment of CE with the pan-PARP inhibitor PJ34^22^ abolished TCDD-enhanced ADP-ribosylation in liver and thymus (Fig. 3a and b), increased NAD^+^ levels in both organs (Fig. 3c and d) and prevented decreased thymus weight and hepatosteatosis by TCDD (Fig. 3e–h and Supplementary Fig. S8). These findings strongly support a role for NAD^+^ depletion in those dioxin toxicities and point to PARP consumption of NAD^+^ as the cause of NAD^+^ depletion by TCDD action. We examined effects of another AhR ligand, β-naphthoflavone (β-NF), on the endpoints affected by TCDD to learn whether our findings were specific for TCDD or applied to other AhR ligands. β-NF activated the AhR in both liver and thymus as evidenced by increased *CYP1A4* mRNA expression, and, like TCDD, suppressed NAD^+^ levels in both liver and thymus and  produced steatosis and thymic atrophy (Supplementary Fig. S9a–d). Treatment with the PARP inhibitor PJ34 prevented hepatosteatosis by β-NF as evidenced by normalized triglyceride levels and curtailed the decrease in thymus weight by β-NF (Supplementary Fig. S9c and d, respectively). The findings show that the effects demonstrated for TCDD can apply also to effects of other AhR ligands.

### Role for decreased Sirt6 activity in TCDD toxicities as a consequence of NAD^+^ loss

**Figure 4:**
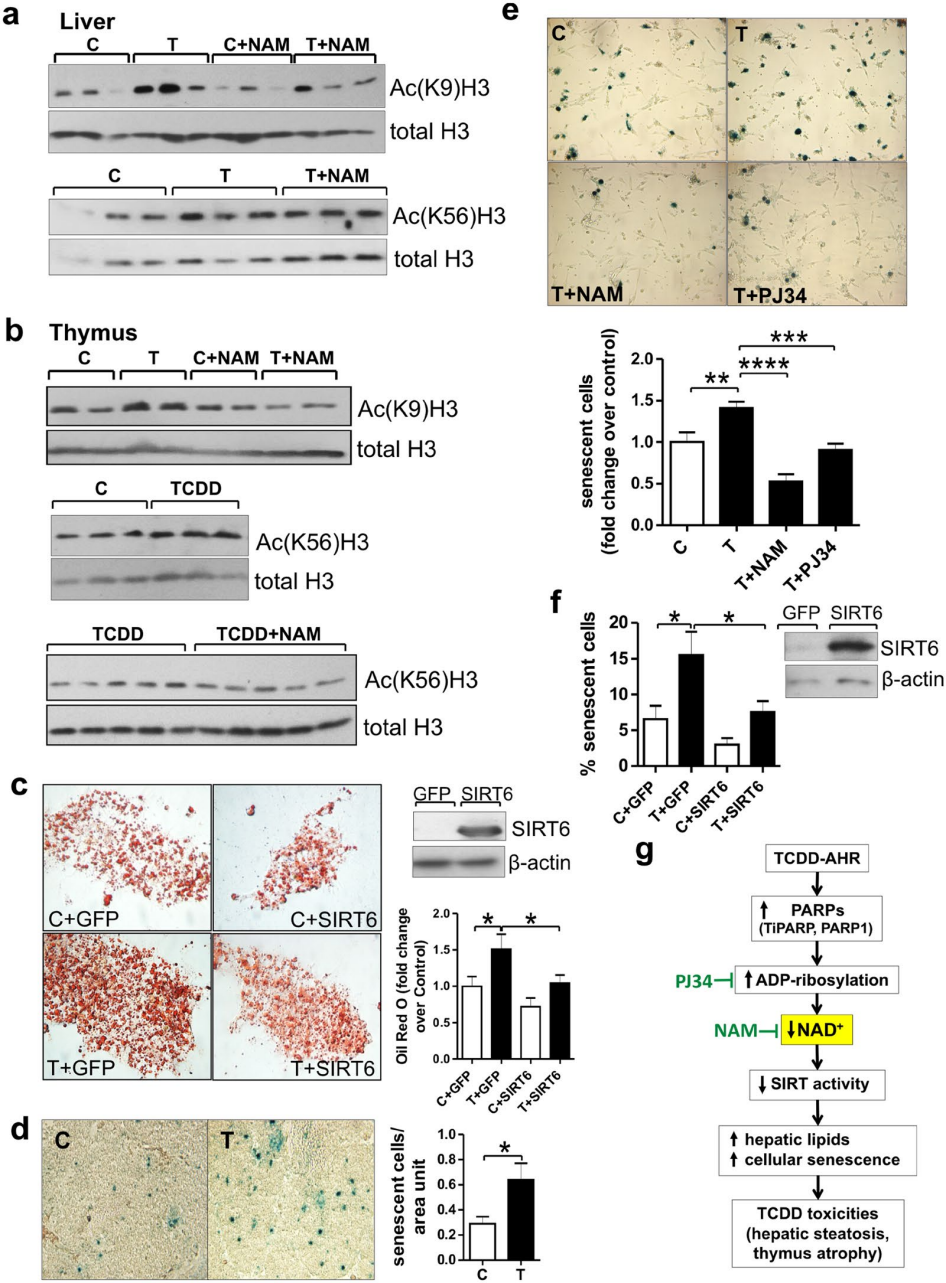
NAD^+^ loss by TCDD decreases Sirt6 activity. Role for Sirt6 in TCDD toxic effects. (**a**) Western blots on homogenates of liver and thymus from CE treated with TCDD or vehicle (n = 3 independent experiments) for 24 hr. CE were cotreated with class I/II deacetylase inhibitors as described in Methods, to allow H3K9 and H3K56 acetylation to serve as indices of Sirt6 deacetylase activity. (**b**) Western blots on homogenates of liver and thymus from CE treated with TCDD (24 hr) with or without NAM for the last 4 hr. (**c**) Representative images of Oil red O stained CEH transfected with an empty plasmid (GFP) or a plasmid containing chick Sirt6 and treated with TCDD (T) or solvent (C). Upper right panel: Western blot on transfected CEH homogenates. (**d**) Representative images for SA-Xgal stained sections of thymus glands from CE treated with TCDD or vehicle for 48 hr. Bar graph shows means ± SE for numbers of senescent cells (blue stained) per unit area of thymus. (**e**) SA-Xgal stained thymic epithelial cells from CE treated with TCDD for 48 hr with or without NAM or PJ34, each injected right after TCDD treatment and 24 hr later. Bar graph shows the mean incidence of senescent cells ± SE per treatment group. (**f**) Means ± SE for incidence of senescent (SA-Xgal stained) thymic epithelial cells transfected with pcDNA-GFP or pcDNA-Sirt6 and treated for 48 hr with TCDD or vehicle. Right panel: Western blot on homogenates of thymic epithelial cells transfected with GFP or Sirt6 plasmids. (**g**) Scheme showing the proposed mechanism by which increased PARP activity produces NAD^+^ loss leading to diverse toxic effects of dioxin *in vivo*.

As changes in NAD^+^ levels affect activities of sirtuins, NAD^+^ dependent enzymes that are major controllers of aging, immune responses and metabolism^20, 23, 24^, we examined effects of NAD^+^ suppression by TCDD on sirtuin activity. We focused on Sirt6 based on reports that Sirt6 knockdown in mouse models was accompanied by thymic atrophy^25^ and hepatic steatosis^26^. TCDD treatment increased acetylation of known Sirt6 deacetylase targets, histone 3 lysine 9 (H3K9) and histone 3 lysine 56 (H3K56)^27^ consistent with TCDD decreasing Sirt6 activity in both organs. NAM treatment prevented those effects (Fig. 4a and b).

In liver, TCDD decreased Sirt6 protein levels as well as Sirt6 activity without affecting *Sirt6* mRNA (Supplementary Fig. S10), consistent with previous evidence that changes in NAD^+^ can affect sirtuin protein levels without changing sirtuin mRNA levels^20^. This effect too was prevented by NAM (Supplementary Fig. S10). Thus decreased Sirt6 protein levels may contribute to decreased Sirt6 activity by TCDD treatment in liver. Overexpression of Sirt6 in CE hepatocytes prevented lipid accumulation by TCDD as evidenced by Oil red O staining (Fig. 4c), consistent with a role for decreased Sirt6 activity in production of hepatosteatosis by TCDD.

Thymus atrophy involves loss of cells and thus may involve cell death. Consistent with the comment of Virag *et al*.^28^ that PARP1 is an infrequent cause of apoptosis, we did not find any consistent evidence for a role for apoptosis in thymus atrophy by TCDD, as evidenced by effects of TCDD on apoptotic indices (i.e. DNA laddering, PARP or caspase cleavage and cytochrome c release from mitochondria to cytosol). The possibility that TCDD may increase the activity of the parthanatos pathway, a cell death pathway related to PARP1 activation and involving AIF (apoptosis inducing factor) translocation from mitochondria to nucleus warrants further investigation^28^.

As Sirt6 suppression has been associated with increased cellular senescence^29^ which could contribute to thymus atrophy by TCDD, we examined effects of TCDD on cellular senescence. TCDD increased cellular senescence in thymus epithelial cells (Fig. 4d,e and Supplementary Fig. S11) and this TCDD effect, like thymus atrophy and hepatosteatosis, was diminished by cotreatment with NAM or PJ34, supporting a role for NAD^+^ depletion by PARP activity in increased cellular senescence by TCDD (Fig. 4e). We found that Sirt6 overexpression diminished the increase by TCDD in cellular senescence in cultured thymic epithelial cells (Fig. 4f), consistent with a role for decreased Sirt6 in this TCDD effect.

## Discussion

The findings reported here identify NAD^+^ loss through PARP activation as a novel unifying mechanism for diverse effects produced by dioxin *in vivo*. Figure 4g presents a scheme for the sequence of events following TCDD exposure based on the findings reported here. Activation of the AhR by TCDD increases PARP activity leading to NAD^+^ loss. Decreased NAD^+^ levels cause different toxic manifestations of TCDD *in vivo* by suppressing NAD^+^- dependent sirtuin activity. Inhibition of PARP activity with PJ34 or supplementation with nicotinamide increases NAD^+^ levels and prevents TCDD toxic effects.

Our findings are consistent with other evidence that PARPs can be involved in the pathogenesis of hepatic steatosis and that PARP inhibition can ameliorate steatosis produced by high fat diet^30–32^. Our report extends those findings by showing that PARP is also involved in steatosis caused by activation of the AhR by toxic chemicals such as dioxin, demonstrating a new role for PARP activity in environmental toxicology.

Several lines of evidence support sirtuin involvement in the downstream effects of AhR activation by TCDD. For example, sirtuin activity is related to NAD^+^ levels in other contexts, becoming suppressed when NAD^+^ levels decline and restored as NAD^+^ is elevated^8, 33^. We focused on Sirt6 because Sirt6 knockdown has been reported to produce effects like those shown here for TCDD. For example, Sirt6 knockdown decreased thymus size^25^, produced steatosis in mouse liver and in hepatocytes^26, 34^ and increased cellular senescence^29, 35^. However, Sirts other than Sirt6 may also be involved. For example, correction of high fat diet-induced hepatosteatosis in mice by nicotinamide riboside was reported to be associated with increased activities of Sirt1 and Sirt3^31^. Suppressed Sirt3 has been associated with hepatosteatosis produced by the constitutively activated AhR in mouse liver^36^. Thus it will be interesting to study effects of each of the remaining six sirtuins on the endpoints shown here.

While increased TiPARP and PARP1 could account for increased ADP-ribosylation by TCDD in liver they might not fully explain increased ADP-ribosylation by TCDD in thymus especially at later times when TiPARP expression declined and PARP1 was not increased. As PARP3, PARP9 and PARP14 have been reported to be prominently expressed in thymus^37, 38^ they are candidates for further exploration of TCDD effects in the thymus.

These studies focus on TCDD as a prototype AhR activator and highly toxic chemical, and also provide evidence supporting NAD^+^ depletion and PARP activity in effects of AhR activation by another AhR ligand, β-NF. It will be interesting to examine the role of NAD^+^ depletion and PARP activity in the effects of the many other environmental contaminants that activate the AhR such as benzo(a)pyrene, PCBs^39, 40^, and cigarette smoke^41^ as well as arsenic, which causes thymic atrophy independently of the AhR^42^.

Some observations reported here point to roles for PARP activity and NAD^+^ in thymus function independently of effects of dioxin or AhR activation, meriting further study. For example, we provide evidence that NAD^+^ levels were an order of magnitude lower in thymus than in liver and that PARP activity (ADP ribosylation) and levels of PARP enzymes (PARP1 and TiPARP) were much higher in thymus than liver in both CE and the mouse. The latter finding is consistent with evidence for low levels of *PARP1* mRNA in mouse liver^43^ and shows further the much larger amounts of PARP and PAR activity in thymus, suggesting that there are undeciphered roles for PARPs and ADP-ribosylation in thymus function.

Thymus atrophy, an inevitable manifestation of human aging, is thought to contribute to increased autoimmunity and decreased responses to infection in aging adults^44^, but there has been surprisingly little attention to forestalling age-related thymus atrophy. The discoveries here that decreased NAD^+^ has a pathogenic role in thymus atrophy by TCDD and that NAD^+^ repletion can prevent thymus atrophy provide a basis for investigating the role of NAD^+^ in thymus atrophy in aging. Further, evidence that declining NAD^+^ levels accompany human aging^45^ provides additional impetus to investigate the contribution of exposure to AhR-activating environmental toxicants to adverse effects of aging.

## Methods

### Chick embryos (CE) and treatments

Fertilized White Leghorn eggs (*Gallus gallus*) (Animal Science Department, Poultry Unit, University of Connecticut) were maintained at 37 °C at high humidity. Fourteen to 18 day old chick embryos (CE) (hatching is at 21 days) were treated *in ovo* by injection through a hole in the shell into the fluids surrounding the embryo, as follows: TCDD, 1 nmol per egg in 0.005 ml dioxane (a dose that produces maximal induction of 7-ethoxyresorufin deethylase (EROD), an index of CYP1A activation^46^); nicotinamide (NAM) (Sigma-Aldrich, St. Louis, MO), 10 mg/egg in 0.05 ml of distilled water; nicotinamide riboside (NR) (gift from Chromadex, Irvine CA), 2 mg/egg in 0.05 ml of distilled water; the pan-PARP inhibitor PJ34 hydrochloride (MedChemExpress, Princeton, NJ), 2 mg/egg in 0.1 ml of distilled water. Unless otherwise indicated, NAM or NR were administered twice (at the same time as TCDD and 36 hr after TCDD treatment) and PJ34 three times (at the same time as TCDD and 24 and 48 hr after TCDD treatment). TCDD treatment was for 72 hr unless otherwise indicated. All controls received vehicle (dioxane or water) in volumes equivalent to those of the treatments. For experiments in Fig. 4a and b CE were treated with suberoylanilide hydroxamic acid (SAHA) (2 mg/egg for the last 4 hr of TCDD treatment) or trichostatin A (TSA) (0.1 mg/egg for last hour) to inhibit classI/II deacetylases, allowing H3K56 and H3K9 deacetylation to serve as indices of Sirt6 activity. Untreated liver samples from 2-week and 10-week old C57BL/6 mice were donated by Dr. Roberto Levi, Weill Cornell Medicine, NY.

### Hematoxylin and eosin (H&E) and Oil Red O (ORO) staining

Pieces of livers and thymus glands removed from CE were promptly placed in 4% paraformaldehyde in 1X PBS at 4 °C for 24 hr fixation, transferred to 30% sucrose for 24 hr, cryoembedded and stored at −20 °C until sectioned (10 μm slices). H&E and Oil red O staining on tissue sections were performed by the Optical Microscopy Core Facility and the Laboratory of Comparative Pathology (Weill Cornell Medicine, NY), following standard procedures. *Oil Red O staining in CEH*: CEH were washed twice with 1X PBS and fixed in 10% formalin for 1 hr at room temperature, then washed twice with distilled H_2_O and incubated in 60% isopropanol for 5 min. After aspirating 60% isopropanol, cells were left to dry at room temperature. One ml of Oil Red O solution (3:2 v/v, Oil Red O stock (3.5 mg/ml isopropanol):dH_2_O) was added to each well and incubated for 10 min. Cells were washed 4 times with dH_2_O. Images were taken using MagnaFire Software on a Nikon Narishige Diaphot microscope and quantified using Image J software (Rasband, W.S., ImageJ, U. S. National Institutes of Health, Bethesda, Maryland, USA, http://imagej.nih.gov/ij/, 1997–2016).

### Triglyceride measurements

Triglycerides were measured using a Triglyceride Colorimetric Assay kit (Cayman Chemicals, Ann Arbor, Michigan) following the manufacturer’s instructions.

### NAD^+^

NAD^+^ levels were measured in liver and thymus homogenates using EnzyChrom NAD^+^/NADH Assay Kits (BioAssay Systems, Hayward CA) following the manufacturer’s directions.

### SDS polyacrylamide gel electrophoresis (PAGE)/Western blotting

Tissue samples were homogenized in 2X sample buffer (125 mM TRIS-HCl, 4% SDS, 16% glycerol, 10% β-mercaptoethanol, 0.002% bromophenol blue) and boiled for 5 min. Proteins were separated on precast Tris-Glycine gels (Invitrogen/Life Technologies) and transferred to nitrocellulose membranes. The following primary antibodies were used: PAR (poly(ADP)ribose) (ENZO, ALX-210-890, lot L29675) which recognizes ADP-ribosylated proteins, TiPARP (custom- made; GenScript, Piscataway, NJ)^7^, PARP1 (Cell Signaling, 9532, lot 7), SIRT6 (Abcam, ab62739; lot GR183923-7), total Histone 3 (H3) (Sigma, H0164, lot 025K4803), acetylated (K9)H3 (Millipore, ABE18, lot 2550911), acetylated (K56)H3 (Cell Signaling, 4243, lot 4), histone H2AX (Abcam 124781, lot GR181357-4), phospho(Ser139)-histone H2AX (Millipore, 05-636, lot 2652964), β-actin (Sigma-Aldrich, A5441, lot 064M4789V). Peroxidase-conjugated goat anti-rabbit (Sigma-Aldrich) or goat anti-mouse (Santa Cruz Biotechnology) antibodies were used as secondary antibodies.

Protein bands were detected using ECL Western Blotting Detection reagents (GE Healthcare, UK). Band intensities were measured by densitometry using Syngene Software (Syngene, Frederick, MD).

### Cell culture

*Chick embryo hepatocytes (CEH)* for the experiments shown in Fig. 4c were cultured as described^7^. To ensure lipid uptake for Oil Red O staining Albumax (1%) (Gibco, Thermo Fisher Scientific, Waltham, MA) was added to the medium when cells were treated with TCDD (1 nM) or vehicle. *Thymic epithelial cells* were cultured as follows: 15–20 thymus glands from CE were placed in 3 ml RPMI 1640 medium containing 2 mg/ml papain and incubated at 37 °C for 15 min, followed by addition of 3 ml RPMI 1640 supplemented with 5% FBS. Thymus glands were chopped with a blade for 3 min and passed through a 100 µm mesh. Cells were plated in 6 cm dishes (about 12 million total thymus cells per dish) in RPMI 1640 supplemented with 5% FBS and 1% pen-strep and allowed to attach for 2 days without changing the medium. On day 3, the medium was replaced with fresh medium, and non-adherent lymphocytes and non-viable cells were removed. The thymic epithelial cells were treated with TCDD at 10 nM.

### Chicken Sirt6 overexpression construct

Chicken Sirt6 was cloned as follows: The chicken Sirt6 coding region (from +47 to +1120 bp) was amplified by PCR with primers designed using the predicted chicken Sirt6 mRNA sequence (Genbank, NM_001039320.1): forward primer, 5′- CACCatggcggtgaattacgcg-3′; reverse primer, 5′-ggtgaggagaggctctacc-3′. cDNA from CE liver was used as a template. The PCR amplification product was purified using Wizard SV Gel and PCR Clean-up System (Promega Co., Madison, WI) and cloned into pcDNA™3.1 using a pcDNA™3.1 Directional TOPO Expression Kit (Invitrogen, Carlsbad, CA) following the manufacturer’s directions. Nucleotide sequencing confirmed the PCR product as chicken Sirt6 (Gene Bank #: KX395903).

### Cell transfection

CEH and thymic epithelial cells (TEC) were transfected using AMAXA Nucleofection technology^7^ using the A023 and T-030 programs, respectively. Transfection efficiency in CEH and TEC was >80% as detected by fluorescence using a plasmid containing GFP.

### Senescence-Associated β-galactoside (SA-β-gal) staining

(a) *Thymus sections:* Thymus glands were removed from CE, fixed in 1X PBS containing 2% v/v formaldehyde and 0.2% v/v glutaraldehyde for 15 min at 4 °C, and incubated in 30% sucrose for 2 hr at 4 °C, then embedded in 30% sucrose:OCT (1:2) and cryosectioned in 10 µm slices. *SA- β-gal assay*: After drying for 30 min, sections were placed in a SA-β-gal staining solution (40 mM citric acid/sodium phosphate solution, pH = 6; 1 mg/ml X-GAL; 5 mM potassium ferrocyanide; 5 mM potassium ferricyanide; 150 mM NaCl; 2 mM MgCl_2_) in a 37 °C incubator with ambient CO_2_. After overnight incubation, blue cells (senescent) were counted using Image J software. (b) *Thymic epithelial cells:* TEC obtained as described above were washed twice with 1X PBS, fixed for 5 min in formaldehyde, washed twice with 1X PBS and placed in the SA-β-gal staining solution. Blue (senescent cells) were counted after overnight incubation at 37 °C. Fixing and SA-β-gal staining solutions were as described above.

### RT-qPCR

Total RNA was extracted from small pieces of liver or thymus and retrotranscribed as reported^7^. For PCR, primers and annealing temperatures were as follows: 18s, 5′-gaccataaacgatgccgact-3′ and 5′-agacaaatcgctccaccaac-3′ (55 °C); TiPARP, 5′-ccagctccagctccaactac-3′, 5′-ctgtaagaacggcatcagca-3′ (57 °C); CYP1A4, 5′-ggacggaggctgacaaggtg-3′, 5′-tgctgcaggatggtggtgag-3′ (59 °C); PARP1 5′-gcaggaaaaacagctgaagg-3′, 5′-gcatcgctcttgaacacaaa-3′(54 °C), Sirt6, 5′- ctactcggataagggcaagt-3′, 5′- cttccatagtccagacacca-3′. An Eppendorf Mastercycler ep gradient machine was used for 40 cycles of amplification. Fold-changes in mRNA were calculated by the standard 2^−ΔΔCt^ method^47^ using 18S mRNA as a reference for normalization.

### Protein concentration

Protein concentration was measured using Biorad-DC or RC-DC Protein assays (Bio-Rad Laboratories, Inc. Hercules, CA).

### Statistics

Differences between group means were evaluated using GraphPad software by unpaired, two tailed t-tests; *p* values ≤ 0.05 were considered statistically significant. All experiments were replicated with the number of independent experiments and numbers of CE in each experiment indicated in the Figure legends.

## Electronic supplementary material

Supplementary Figures 1–11
