# Supplementary material for: NAD+ loss, a new player in AhR biology: prevention of thymus atrophy and hepatosteatosis by NAD+ repletion
Source: Sci Rep. 2017 May 23;7:2268. doi: 10.1038/s41598-017-02332-9 (PMC5442136; doi:10.1038/s41598-017-02332-9)
Supplement: Supplementary file 1 — Supplementary Figures 1–11 [file 41598_2017_2332_MOESM1_ESM.pdf]

Supplementary Information

NAD$^+$ loss, a new player in AhR biology: prevention of thymus atrophy and hepatosteatosis by NAD$^+$ repletion.

Silvia Diani-Moore[1], Jenny Shoots[1], Rubi Singh[1], Joshua B. Zuk[1] and Arleen B. Rifkind[1*]

[1]Department of Pharmacology and Pharmacology PhD Program, Weill Cornell Medicine, 1300 York Avenue, NY, NY, 10021.

* *Corresponding Author:* Arleen B. Rifkind, MD. [1]Department of Pharmacology, LC401, Weill Cornell Medicine, 1300 York Avenue, NY, NY, 10021.

Email address: arifkind@med.cornell.edu. Telephone: 212-746-6236. FAX: 212-746-8835

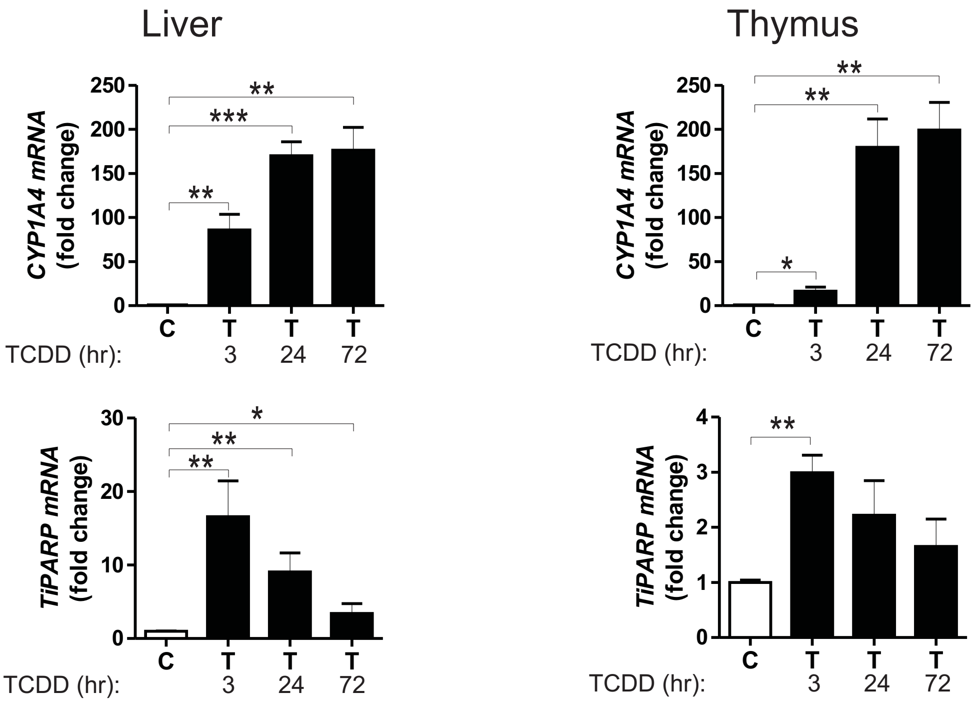

Supplementary Figure S1. TCDD increases *CYP1A4* and *TiPARP* mRNA levels in liver and thymus. Mean fold change in mRNA levels ± SE for *CYP1A4* and *TiPARP* in livers and thymus glands from CE treated with TCDD or vehicle for 3, 24 and 72 hr (n=3-9 CE per treatment group).

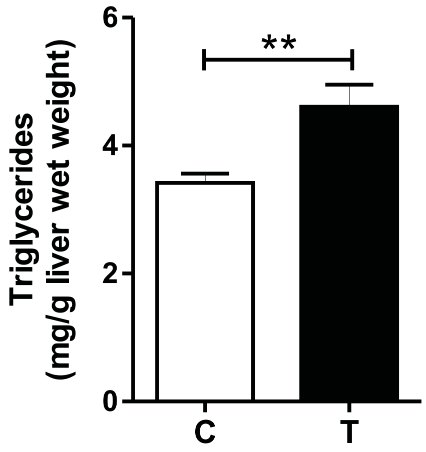

Supplementary Figure S2. TCDD increases hepatic triglycerides. Mean triglyceride levels ± SE for livers from chick embryos (CE) 72 hr after treatment with TCDD (T) or dioxane (C) (n= 8 CE per treatment group).

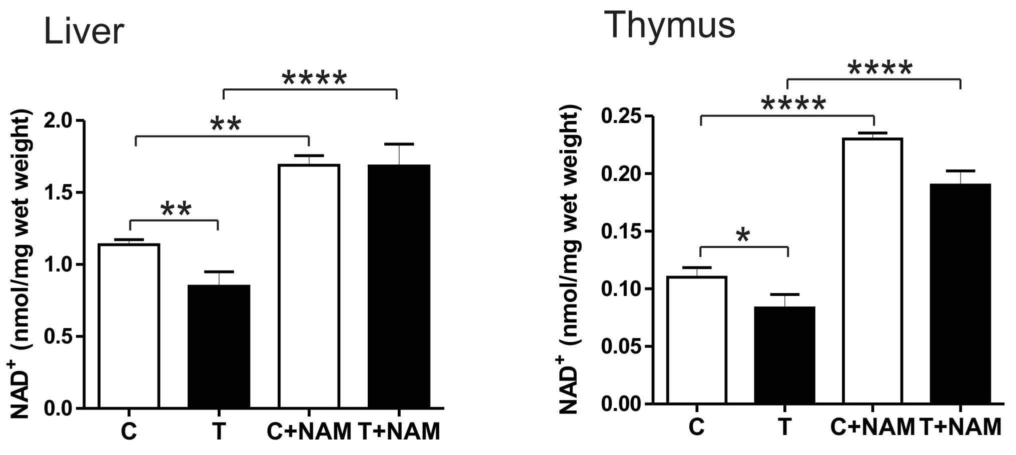

Supplementary Figure S3. A second dose of NAM prolongs NAD$^+$ elevation in both thymus and liver. CE were treated with TCDD with or without NAM 24 and 48hr later. NAD$^+$ levels at 4 hr after the second administration of NAM are shown. Bar graphs show means ± SE.

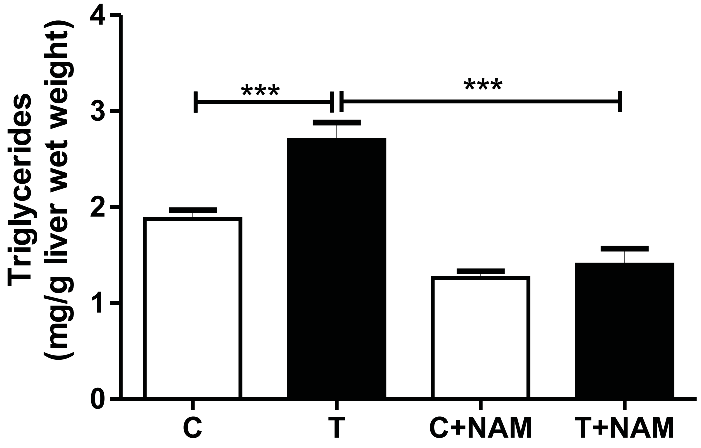

Supplementary Figure S4. NAM administration prevents increased liver triglycerides by TCDD.
Mean triglyceride levels ± SE for livers from CE 72 hr after treatment with TCDD (T) or dioxane (C) with or without NAM administration (n=5-10 livers per treatment group).

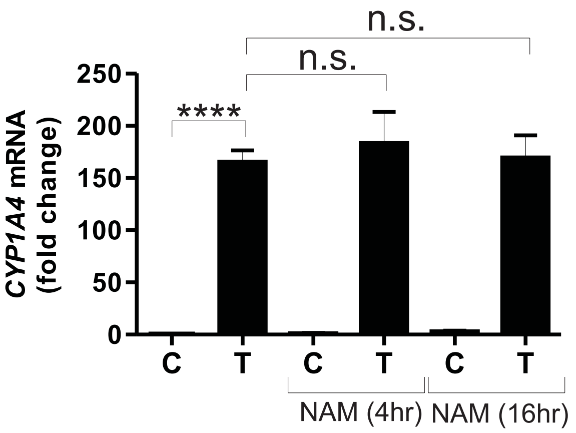

Supplementary Figure S5. NAM administered to CE *in vivo* at 10 mg/egg does not inhibit AhR action. Bar graph shows mean *CYP1A4* mRNA levels ± SE for livers of CE treated with TCDD for 24 hr with or without NAM for the last 4 or 16 hr (n=3-5 CE per treatment group).

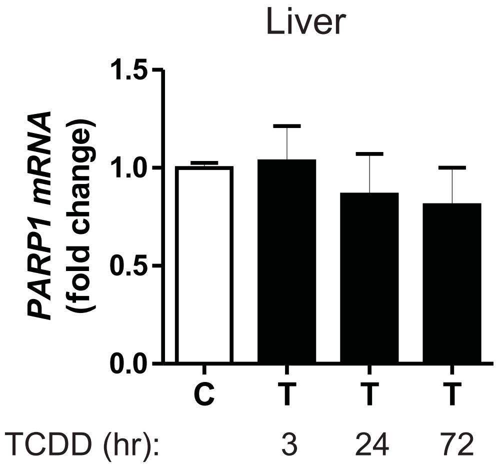

Supplementary Figure S6. TCDD does not affect *PARP1* mRNA levels. Mean *PARP1* mRNA levels ± SE are shown for livers of 18-day old CE after treatment with TCDD for 3, 24 and 72 hr (n=3-9 individual livers per treatment group).

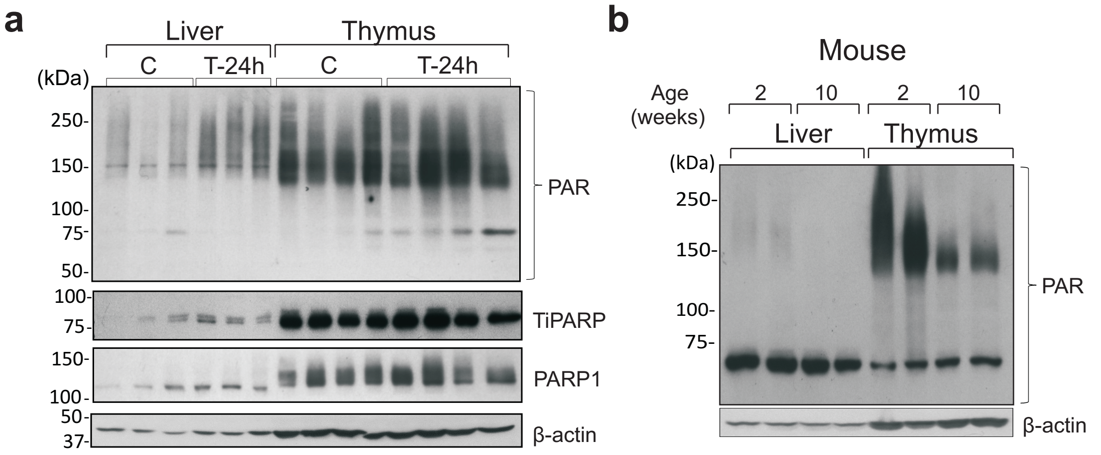

Supplementary Figure S7. Thymus has higher levels of ADP-ribosylation, TiPARP and PARP1 than liver. a. Western blots on homogenates of livers and thymus glands from Control and TCDD-treated CE, on the same nitrocellulose membrane (30 µg protein per lane). **b**. Western blots on homogenates of livers and thymus glands from untreated 2- and 10-week old C57BL/6 mice.

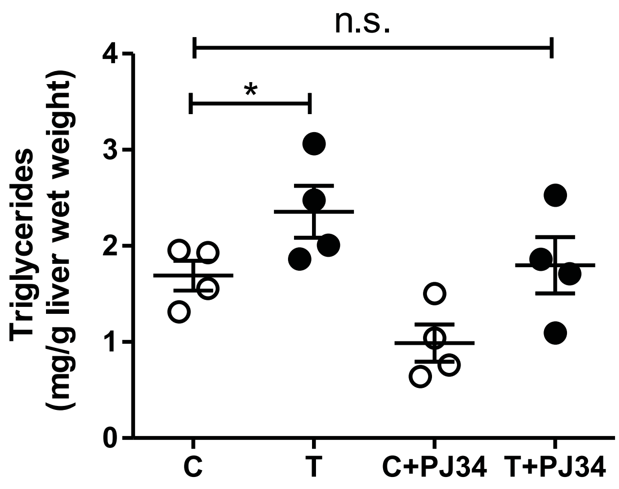

Supplementary Figure S8. PARP inhibition by PJ34 prevents increased liver triglycerides by TCDD. Mean triglyceride levels ± SE for livers from CE 72 hr after treatment with TCDD (T) or dioxane (C) with or without the PARP inhibitor PJ34 (n=4 CE per treatment group).

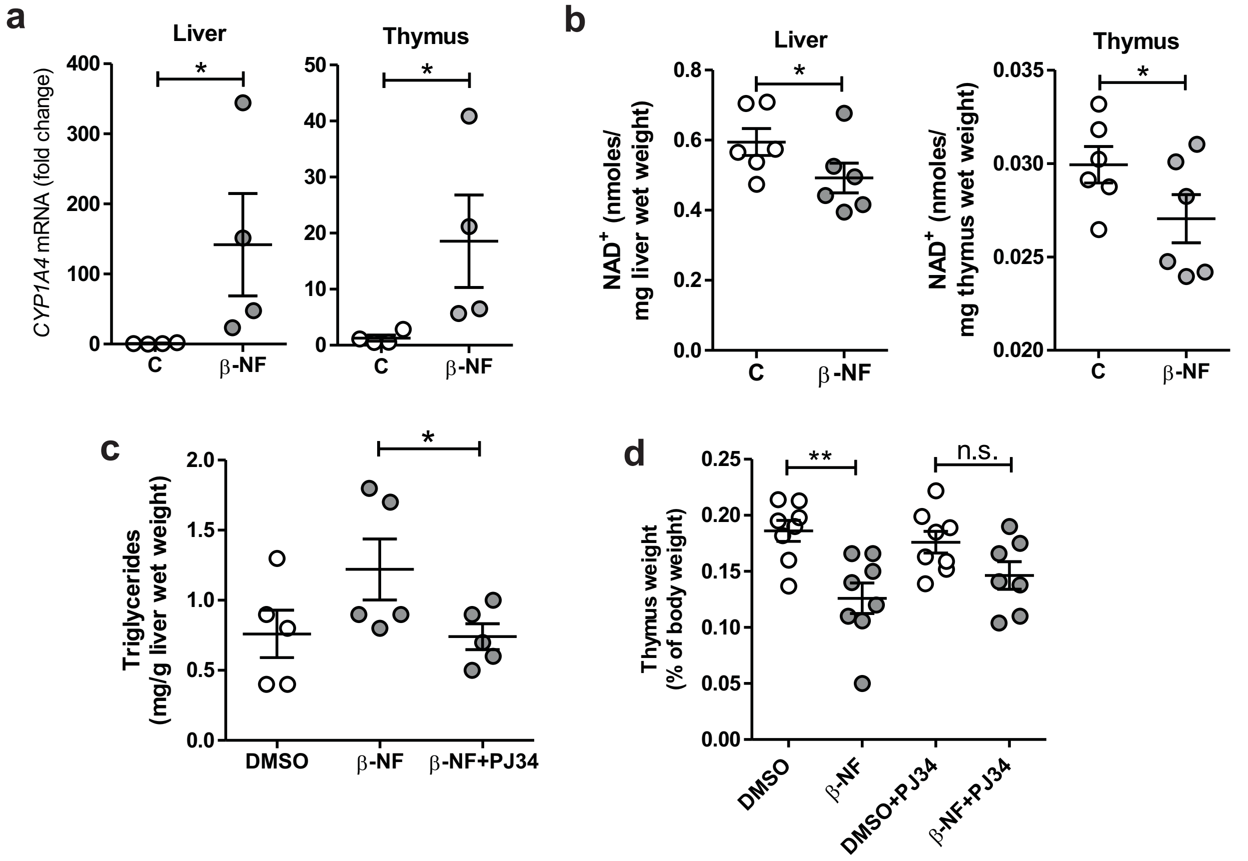

Supplementary Figure S9. β-NF increases *CYP1A4* mRNA and decreases NAD+ levels in both liver and thymus. β-NF increases hepatic triglycerides and decreases thymus weight, effects prevented by the PARP inhibitor PJ34. CE were treated for 72 hr with β-naphthoflavone (β-NF) (6.7 mg/egg) or the solvent DMSO. **a.** Mean fold change ± SE for *CYP1A4* mRNA levels in livers and thymus glands; **b.** Means ± SE for NAD+ levels in livers and thymus glands. **c.** and **d.** CE were treated as above with or without the PARP inhibitor PJ34. Means ± SE for hepatic triglycerides levels and for thymus weights as percentages of body weight are shown.

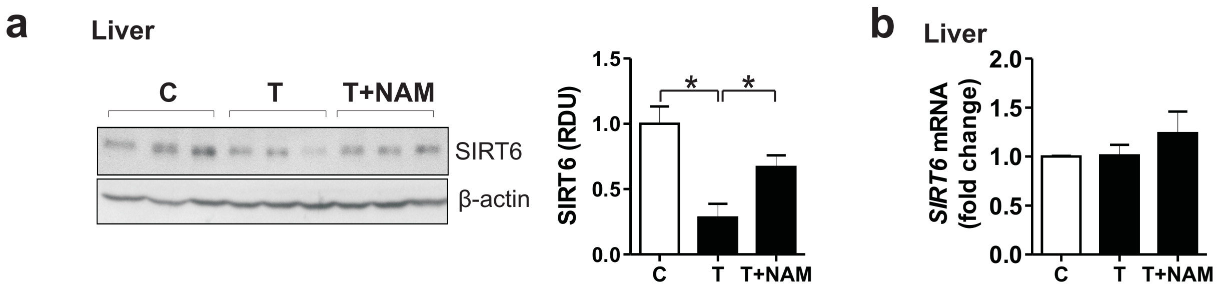

Supplementary Figure S10. TCDD decreases hepatic Sirt6 protein levels without affecting *Sirt6* mRNA; NAM prevents decrease in Sirt6 levels by TCDD. CE were treated for 24 hr with TCDD or vehicle with or without NAM overnight. **a.** Western blot for Sirt6 protein for controls and CE treated with TCDD or TCDD + NAM (n=4 independent experiments). **b.** Lack of effect of the treatments on *Sirt6* mRNA levels (n=5 independent experiments). Data are expressed as means ± SE.

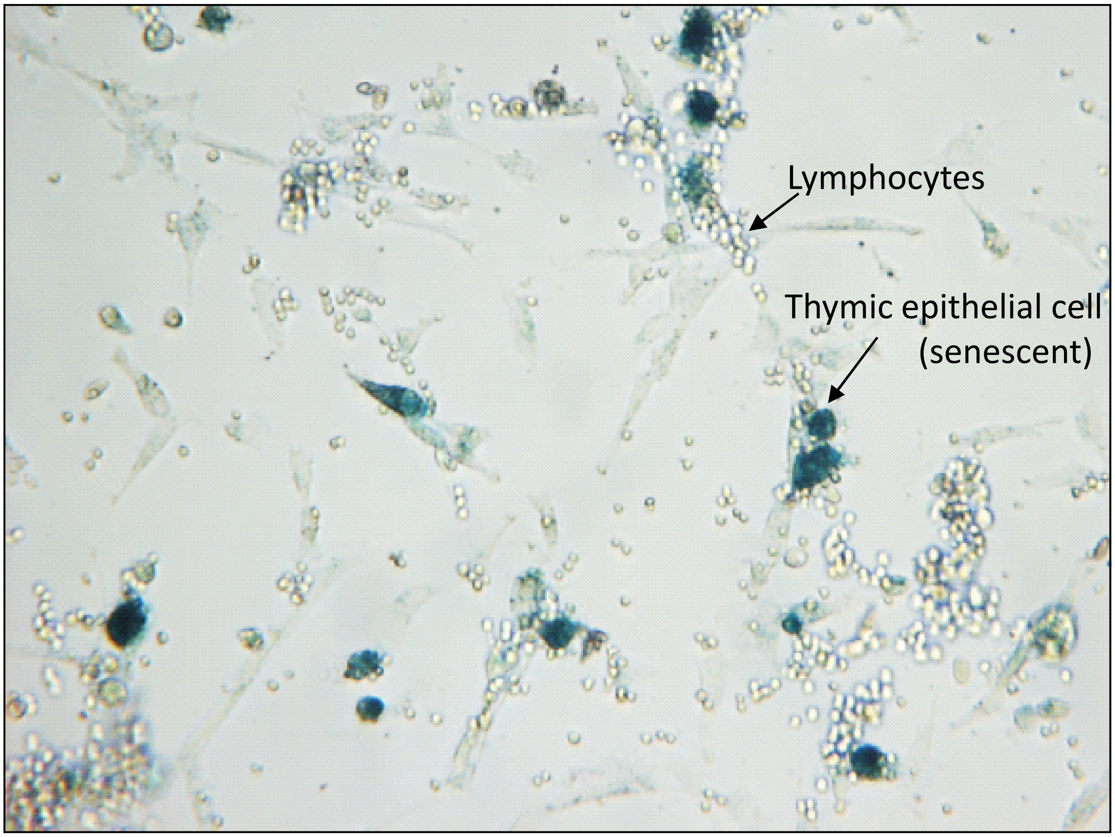

Supplementary Figure S11. Thymic epithelial cells but not lymphocytes stain for senescence using SA-Xgal. Cultured epithelial cells from thymus glands of 17-day old CE after CE were treated with TCDD for 48 hr. Senescence (blue staining) was seen only in epithelial cells and not in lymphocytes (smaller round cells).
